# A Synthetic Microbiome Based on Dominant Microbes in Wild Rice Rhizosphere to Promote Sulfur Utilization

**DOI:** 10.1186/s12284-024-00695-y

**Published:** 2024-03-01

**Authors:** Changji Wang, Jingjing Chang, Lei Tian, Yu Sun, Enze Wang, Zongmu Yao, Libo Ye, Hengfei Zhang, Yingnan Pang, Chunjie Tian

**Affiliations:** 1 State Key Laboratory of Black Soils Conservation and Utilization, Northeast Institute of Geography and Agroecology, Chinese Academy of Sciences, Changchun, 130102 China; 2 Key Laboratory of Mollisols Agroecology, Northeast Institute of Geography and Agroecology, Chinese Academy of Sciences, Changchun, 130102 China; 3 University of Chinese Academy of Sciences, Beijing, 100049 China; 4 College of Resources and Environment, Jilin Agricultural University, Changchun, Jilin China; 5 College of Life Science, Jilin Agricultural University, Jilin Changchun, China

**Keywords:** Rice domestication, Rhizosphere microbiome, Sulfur cycling, Dissimilatory sulfate reduction, Synthetic microbiome

## Abstract

Sulfur (S) is one of the main components of important biomolecules, which has been paid more attention in the anaerobic environment of rice cultivation. In this study, 12 accessions of rice materials, belonging to two Asian rice domestication systems and one African rice domestication system, were used by shotgun metagenomics sequencing to compare the structure and function involved in S cycle of rhizosphere microbiome between wild and cultivated rice. The sulfur cycle functional genes abundances were significantly different between wild and cultivated rice rhizosphere in the processes of sulfate reduction and other sulfur compounds conversion, implicating that wild rice had a stronger mutually-beneficial relationship with rhizosphere microbiome, enhancing sulfur utilization. To assess the effects of sulfate reduction synthetic microbiomes, Comamonadaceae and Rhodospirillaceae, two families containing the genes of two key steps in the dissimilatory sulfate reduction, *aprA* and *dsrA* respectively, were isolated from wild rice rhizosphere. Compared with the control group, the dissimilatory sulfate reduction in cultivated rice rhizosphere was significantly improved in the inoculated with different proportions groups. It confirmed that the synthetic microbiome can promote the S-cycling in rice, and suggested that may be feasible to construct the synthetic microbiome step by step based on functional genes to achieve the target functional pathway. In summary, this study reveals the response of rice rhizosphere microbial community structure and function to domestication, and provides a new idea for the construction of synthetic microbiome.

**Supplementary Information:**

The online version contains supplementary material available at 10.1186/s12284-024-00695-y.

## Introduction

Sulfur (S) has garnered significant attention in recent years due to its biological functions, especially in rice. It plays a vital role in the synthesis of proteins, vitamins, and other biomolecules which are necessary in plant physiological processes (Leustek et al. 2000). The primary sulfur compound assimilated by plants is sulfate (SO_4_^2−^) (Kopriva et al. 2019). Sulfate is reduced to cysteine through assimilatory sulfate reduction in plants, and then form disulfide bonds to participate in protein formation (Leustek et al. 2000). Important sulfur compounds based on assimilatory sulfate reduction, such as glutathione, phytochelatins and metallothionein, are involved in physiological processes such as antioxidant reaction, resistance to heavy metal stress and phytochemicals protecting plants against pathogens and herbivory (Takahashi 2019). Hydrogen sulfide (H_2_S), an important gas signaling molecule (Arif et al. 2021), is different from sulfate which can be absorbed directly. Hydrogen sulfide can promote seed germination (Chen et al. 2017), root growth (Li et al. 2014a) and other important plant life processes. At the same time, it also can stimulate plant cells, and then make plants resistant to abiotic stresses such as drought (Jin et al. 2018), salt (Jiang et al. 2019), heat (Li et al. 2014b), cold (Du et al. 2017) and heavy metal stresses (Kaya et al. 2020; Chen et al. 2017; Luo et al. 2020). In the field of agroecology, there exists a strong association between sulfate and hydrogen sulfide, particularly in the context of rice cultivation, with the enclosed environment of paddy field serving to further reinforce this relationship. Microbiome plays a powerful role in this relationship, especially rhizosphere microbiome which mediates the sulfur cycling between soil and plants (Chaudhary et al. 2023).

Rhizosphere microbiome, referred to as the “second genome” of plants, regulates plant growth and development (Lu et al. 2018). It facilitates nutrient uptake, enhances stress tolerance (Berendsen et al. 2012), and serves as a key driver of the soil sulfur cycle, mediating the transformation of sulfur between various forms in the soil (Chaudhary et al. 2023). Gahan and Schmalenberger (2014) have reviewed the significance of rhizosphere microbe on plant sulfur supply, including providing sulfur for plant growth, mineralization of soil organic sulfur. Moreover, researchers have demonstrated that the introduction of specific functional rhizosphere microbiome can influence crop traits (Trivedi et al. 2020; Mueller and Sachs 2015). In the study of Castrillo et al. (2017), specific microbial communities were employed to improve plant phosphorus uptake. According to Zhou et al. (2020), a functional microbiome was linked to support tomato suppression of fusarium wilt disease. As such, using a synthetic microbiota is one of the most effective ways to artificially regulate the rhizosphere microbiome to influence plant growth, which may provide us with the opportunity to promote sulfur utilization in rice. However, the acquisition of members in synthetic microbiome is the primary concern at present.

Previous studies have found that wild relatives have richer rhizosphere microbiome interactions than domesticated crops, which made plants benefit from interaction with the microbiome for better nutrient uptake and greater stress resistance (Gutierrez and Grillo 2022). The rhizosphere microbiome offers diverse support for the growth and development of wild plants (Isaac et al. 2021). Numerous studies have implied that with the aid of rhizosphere microbiome, wild plants exhibit enhanced resistance to drought, pest, pathogen stress and other stressors (Gutierrez and Grillo 2022; Whitehead et al. 2017; Koziol et al. 2012). As a wild relative of cultivated rice, wild rice also engages in more advantageous interactions with the rhizosphere microbiome (Chang et al. 2022; Tian et al. 2022), which could be a way to achieve functional microbes.

In recent years, the high-throughput technology has been applied to obtain strains in batches (Zhang et al. 2021). With high-throughput screening and identification of rhizosphere microbe, we can obtain as many culturable microbe as possible in the rhizosphere microbiome of wild rice, laying a strain resource foundation for the construction of synthetic microbiome.

Although the rhizosphere microbiome of wild rice may have a positive effect on sulfur utilization in cultivated rice, there is few studies verify this relationship explicitly. This study aimed to reveal the differences of sulfur utilization between wild and cultivated rice rhizosphere microbiome, and explore how to construct a synthetic microbiome effectively to improve sulfur utilization in rice.

## Materials and Methods

### Plant Materials

In order to elucidate the changes of rhizosphere sulfur-cycling in rice domestication, twelve accessions of rice materials, which belong to two major rice domestication systems in Asia and Africa that are widely cultivated around the world, were obtained from Jiangxi Academy of Agricultural Sciences, China and International Rice Research Institute (IRRI), Philippines. Materials in the African domestication system include African wild rice IRGC 106,238 (*Oryza barthii*) and African domesticated rice NO. 2, NO. 3 and NO. 4 African domesticated rice (*O. glaberrima*). And there are two branches in Asian rice domestication systems accessions were: Common wild rice IRGC 106,452 and IRGC 106,286 (*O. rufipogon*) and rice japonica Jiangxi and Daohuaxiang (*O. sativa subsp. japonica*), Indian wild rice IRGC 86,655 and IRGC 88,949 (*O. nivara*) and rice indica 106 and Meitezhen (*O. sativa subsp. indica*) (Table 1).

**Table 1:** Rice materials

		Wild rice	Cultivated rice
African		IRGC 106,238	NO. 2, NO. 3, NO. 4
Asian	Indian	IRGC 86,655, IRGC 88,949	Indica 106, Meitezhen
	Common	IRGC 106,452, IRGC 106,286	Japonica Jiangxi and Daohuaxiang

### Site Description and Sample Collection

Our study was conducted at Hainan Rice Breeding Center (18°19 ‘57 N, 109°27’ E), Jiangxi Academy of Agricultural Sciences, China. Wild rice and cultivated rice were grown under the same soil and climate conditions to avoid the effects of soil and environment differences. Soil characteristics and experimental design were described in a comparative study of rhizosphere fungal communities of wild and cultivated rice (Chang et al. 2021).

Five replicates were obtained from each accession during the flowering stage of rice. Five to seven rhizosphere samples were mixed as a biological replicate. (Tian et al. 2022). The adhered soil loosely on roots was shook off, ensuring a residual soil layer about 1 mm thickness remained as the rhizosphere soil sample. Then the 1 mm of soil, which attached to root tightly, was collected by immersing the roots in 5 mL of sterile water and subjecting them in vortex (Edwards et al. 2015). Each rhizosphere soil sample was divided into two parts after a brief centrifugation and removal of the supernatant and stored at 4℃ and − 80℃, respectively.

### DNA Extraction

According to the instructions of the Fast DNA SPIN Kit (MPBio, Santa Ana, USA), DNA was extracted from each 0.5 g rhizosphere soil sample which was pulverized into powder in liquid nitrogen. And then the concentrations were measured with the NanoDrop 2000 Spectrophotometer (Thermo Fisher Scientific, Waltham, USA). The quality of the DNA was detected by 1.2% agarose gel electrophoresis and only the DNA with a clear band was selected for further sequencing. All DNA extracted from the rhizosphere was used for metagenome shotgun sequencing. Each of the DNA fragments had an adapter for DNA library preparation by PCR. The DNA libraries were subjected to paired-end shotgun sequencing using an Illumina HiSeq X TEN platform (San Diego, CA, USA). Tian et al. (2022) have reviewed shotgun metagenomic sequencing and data analysis in great detail explained. Under the bioproject number PRJNA632564 described by Tian et al. (2022), the sequences of 12 wild and domesticated rice accessions with five replicates, totalling 59 samples, were deposited in the National Center for Biotechnology Information (NCBI; https://www.ncbi.nlm.nih.gov/).

### Shotgun Metagenomics Sequencing and Bioinformatics Analysis

According to Chang et al. (unpublished data), shotgun metagenomic sequencing and data analysis has been described in detail. SqueezeMeta pipeline in seqmerge mode was used to process the shotgun metagenomics sequences (Tamames and Puente-Sánchez 2019). Trimmomatic v 0.39 was utilized to remove adapters, trim, and perform quality filtering (Bolger et al. 2014). Megahit v 1.2.9 was used to assemble each sample and contigs were filtered with prinseq (Schmieder and Edwards 2011; Li et al. 2015). For the purpose of ORF prediction and rRNA gene sequence retrieval, Prodigal and barrnap were utilized, respectively (Hyatt et al. 2010; Seemann 2014). For the kyoto encyclopedia of genes and genomes (KEGG) orthology (KO) numbers, taxonomic classification of the ORFs was performed using Diamond software against the most recent publicly accessible version of the KEGG database (Kanehisa and Goto 2000). The S-cycling genes were identified and annotated using the KEGG automatic annotation server. SqueezeMeta script SQM2tables.py was used to calculate the average coverage and normalized TPM (transcripts per million) values for data on gene and function abundances. As a result, we assessed treatment differences as variations in the percentage of functional genes. For the assembled contigs, taxonomic assignment of the N-cycling genes and normalized TPM SqueezeMeta ORF related to nitrogen metabolism dataset were carried out at ORF level, and the outcomes of the MGNify analysis were utilized for statistical analyses carried out in RStudio version 1.1.423 utilizing R 4.1.2 (Team 2022). Concoct, Maxbin, and Metabat were used to binning quality-controlled contigs larger than 1000 kb (Alneberg et al. 2014; Wu et al. 2014; Kang et al. 2015). The DAS tool was then used to refine the resulting bins [40], and dRep was used to perform genome dereplication (Sieber et al. 2018). CheckM was used to verify the taxonomy assignment, completeness, and contamination of the assembled genomes (Olm et al. 2017). Prodigal (Hyatt et al. 2010) was used to predict the genomes’ ORFs, and Diamond (Buchfink et al. 2015) was utilized to functionally annotate the genomes with eggNOG (KO numbers) (Huerta-Cepas et al. 2016).

### Data Analyses and Visualization

Pairwise Spearman rank correlation was used to determine the correlations of rhizosphere microbiome involved in the sulfur-cycling. To visualize co-occurrence networks, Gephi v0.9.2 and Cytoscape v3.9.1 were used to display only significant correlations (Spearman’s|cor| > 0.9, *p* < 0.01) (Bastian et al. 2009; Shannon et al. 2003). Relative abundance analysis and principal components analysis (PCA) were performed on Tutools platform (https://www.cloudtutu.com), a free online data analysis website.

### Dissimilatory Sulfate Reduction Bacteria Isolation

The dominant culturable strains of the rhizosphere were obtained by high-throughput cultivation and identification from the soil of the wild rice rhizosphere (Zhang et al. 2021). The bacteria containing *aprA* and *dsrA*, a gene involved in dissimilatory sulfate reduction, were chosen through PCR functional gene verification using the primers *dsrA*-F (5’-AGCACGTGGTATTGTTTCTG − 3’) and *dsrA*-R (5’-CCGCTTCCATATTTCTCCC − 3’), *aprA*-F (5’-TCACCAATATCCACTTCGTCG − 3’) and *aprA*-R (5’-AGCGCATCCGGTACATCC − 3’).

### Synthetic Microbiome Construction and Functional Testing

All functional strains were propagated using the shake flask fermentation method in postgate medium with 20 h at 37 °C, 150 rpm. Each of the bacterial fermentation broth was centrifuged at 4000×g for 15 min, washed with PBS, and adjusted OD_600_ to 0.02 (∼ 10^7^ cells/mL) (Zhou et al. 2022). The diluted bacterial fermentation broth, containing strains from the same family, was mixed in equal proportions constructed as the mixed bacterial broth of this family. To assess the effect of different component ratios on the synthetic microbiome, three types of synthetic microbiomes were constructed using varying ratios of 8 to 2, 1 to 1, and 2 to 8 of the two families mixed bacterial solution.

Sterilized wild rice seeds were planted in gamma-ray sterilized paddy soil that has been combined with three different synthetic microbial groups respectively at the seedling stage, with sterile soil serving as a control group.

Sterilized cultivated rice seeds were planted in natural paddy soil, which was combined with three different synthetic microbial groups respectively at the seedling stage, without adding the synthetic microbiome as a control group.

The experimental period ran for 7 days, during which there were 15 h of light, a temperature of 35℃, and 9 h of darkness, a temperature of 30℃ with 80% humidity. Each group was treated with 5 pots of replicates. Root length was measured on day 1 and day 7.

## Results

### Differences in S-cycling Rhizosphere Microbiome Between Wild and Cultivated Rice

**Fig. 1:**
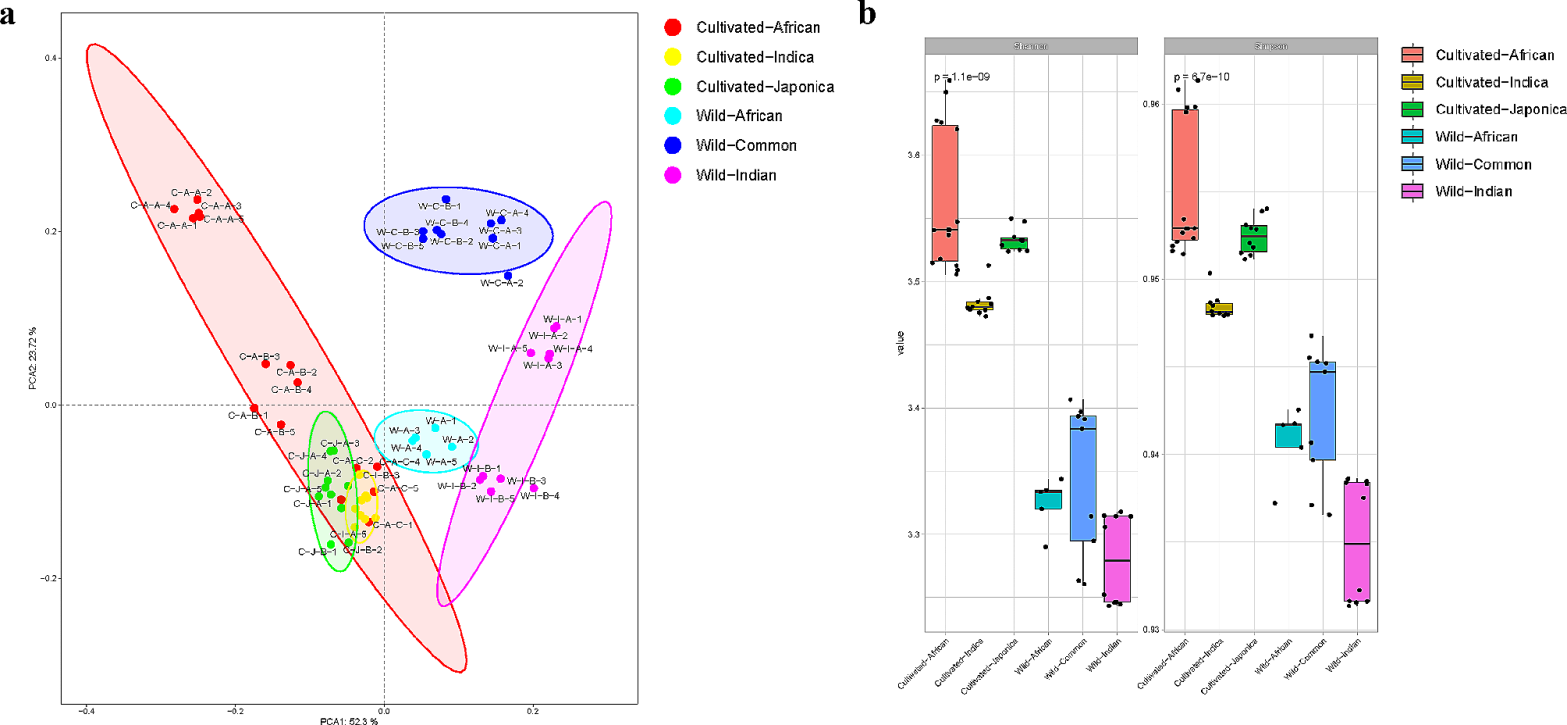
Principal Component Analysis and alpha diversity of S-cycling microbiome in wild and cultivated rice rhizosphere. a A Principal Component Analysis (PCA) plot showed the distribution of S-cycling genes in wild and cultivated rice. b For alpha diversity richness and evenness, Shannon and Simpson indices are presented

As seen in Fig. 1a, Principal Component Analysis (PCA) of S-cycling genes revealed that the wild and cultivated rice rhizosphere samples were distinct in sulfur cycle function gene composition. For the rhizobacterial community, t-test applied to Shannon index showed that the evenness of the microbiome in cultivated rice rhizosphere was significantly higher than that in wild rice rhizosphere, while the Simpson index showed that the community diversity in wild rice rhizosphere was significantly higher than that in cultivated rice rhizosphere (Fig. 1b, *p* < 0.01).

### Differences in Community Composition of S-cycling Rhizosphere Microbiome Between Wild and Cultivated Rice

**Fig. 2:**
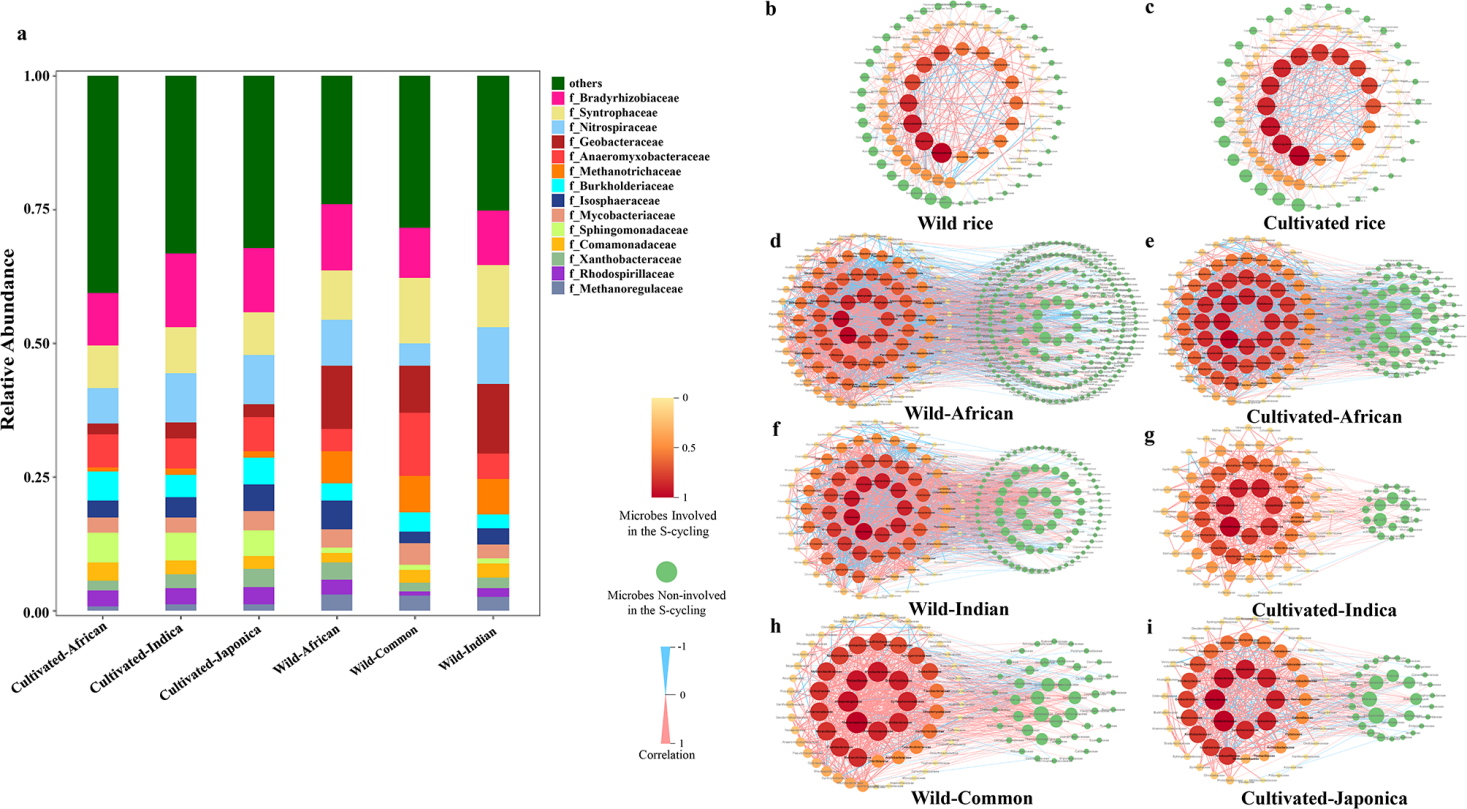
Relative abundance and correlation network of rhizosphere microbiome associated with the S-cycling in wild and cultivated rice. a Relative abundance of wild and cultivated rice rhizosphere microbiome in three domestication systems. b–i Correlation network of wild and cultivated rice rhizosphere microbiome in total and three domestication systems, respectively. Edges represent significant correlations (Spearman’s ρ > 0.9, p < 0.01, light red; Spearman’s ρ < −0.9, p < 0.01, light blue). Nodes with a yellow to red gradient represent microbes that participate in the S-cycling. The darker the color, the greater the correlation. The green nodes represent microbes that do not participate in the S-cycling. b, c Correlation network of rhizosphere microbiome in total wild and cultivated rice, respectively. d, e Correlation network in African domestication system. f, g Correlation network in Indica domestication system. h, i Correlation network in Japonica domestication system

Relative abundance of the top 15 microbial groups at the family level were displayed in Fig. 2a. It was compared how the three domestication systems affected the microbial community structure related to the rhizosphere S-cycling in wild rice versus cultivated rice. In the rhizosphere relative abundance of the three domestication systems, it was discovered that the top 15 microbial groups among the 102 families of microorganisms were the same 15 families with different priorities. More *Syntrophaceae*, *Geobacteraceae*, *Methanobacteriaceae*, *Methanoregulaceae* were found in greater abundance in the rhizosphere of the three wild rice species. Recruiting *Bragyrhizobiaceae* and *Sphingomonadaceae* was the main focus of the cultivated rice rhizosphere. Diverse wild and cultivated rice varieties also offer unique benefits when it comes to attracting different bacterial groups. Common wild rice has a higher abundance of *Anaeromyxobacteraceae* enriched in the rhizosphere, and African wild rice is able to collect more *lsosphaeraceae*.

Correlation network (Fig. 2b–i) was utilized to display the correlations between wild and cultivated rice rhizosphere microbiome of different domestication systems in the S-cycling. Table 2  was integrated by quantifying the nodes and edges in each network. The nodes were the members associated with the S-cycling, and their correlations were represented by the number of edges. Significant variations were observed between wild and cultivated rice in all domestication systems as shown in correlation network. Moreover, the microbe correlations were more complex in the rhizosphere of wild rice (African: 93 + 211 nodes, the number of microbe involved and non-involved in S-cycling, and 2114 edges; Indian: 84 + 89 nodes and 1760 edges; Common: 67 + 44 nodes and 724 edges) than cultivated rice (African: 82 + 78 nodes and 1498 edges; Indica: 70 + 21 nodes and 311 edges; Japonica: 59 + 31 nodes and 487 edges). During the maximum and median of degrees, it was also found that wild rice rhizosphere had more highly correlated microbe than cultivated. In contrast, the integration of different domestication systems showed that the rhizosphere microbial correlation index of wild rice decreased more significantly than that of cultivated rice, which may be due to the more significant difference between wild rice in different domestication systems (Table 2).

**Table 2:** Quantitative index of microbial correlation network

	Degrees			Microbes involved in the S-cycling	Microbes non-involved in the S-cycling
	Maximum	Median	Total		
African wild rice	78	36	2114	93	211
African cultivated rice	53	27	1498	82	78
Indian wild rice	73	30	1760	84	89
Indica cultivated rice	19	6	311	70	21
Common wild rice	44	9	724	67	44
Japonica cultivated rice	35	7	487	59	31
Total of wild rice	20	6	282	56	41
Total of cultivated rice	30	6	369	60	31

### Differences in Metabolic Pathways of Rhizosphere Microbiome Between Wild and Cultivated Rice

**Fig. 3:**
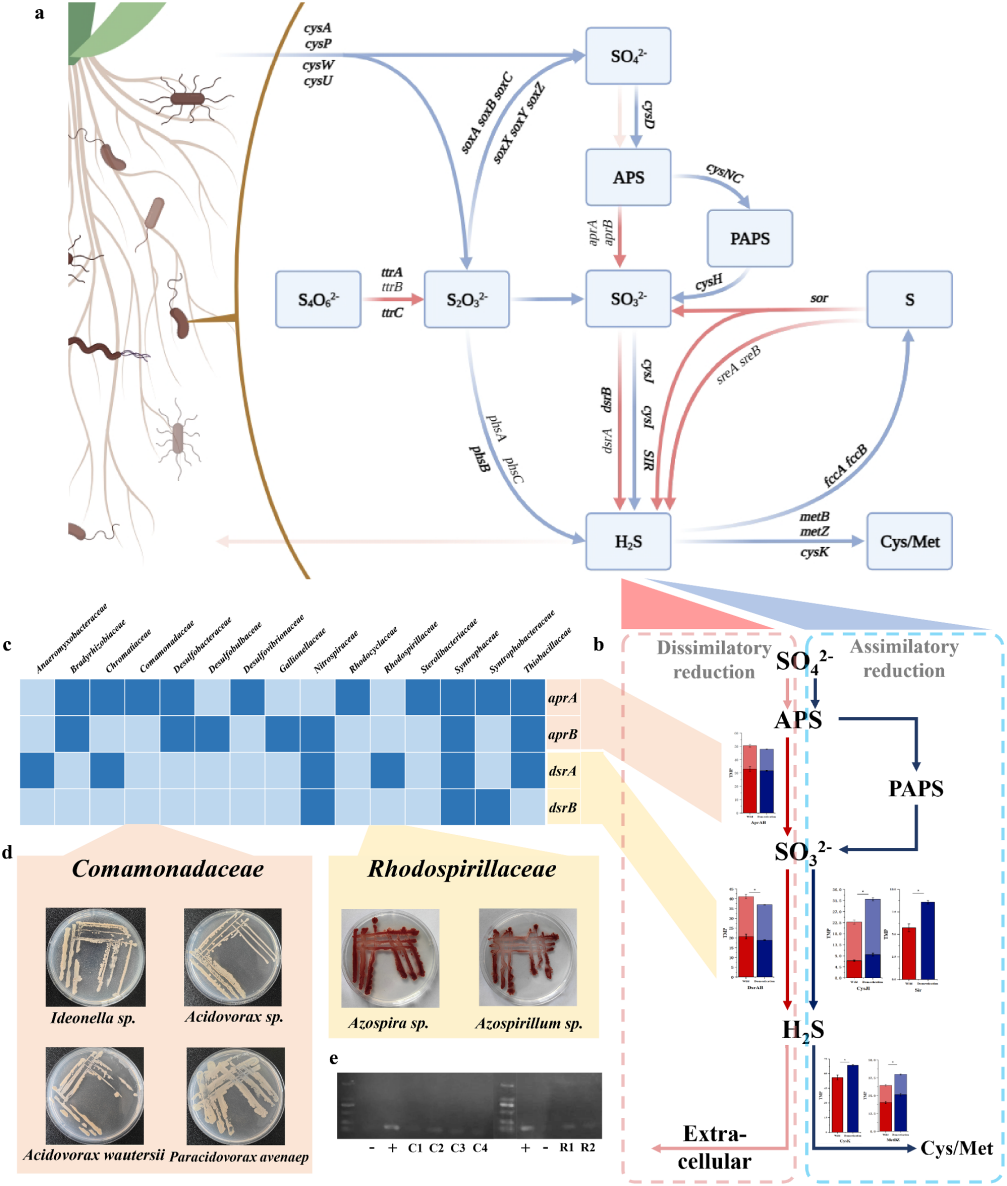
Differences in S-cycling metabolic pathways of wild and cultivated rice rhizosphere microbiome, and isolation of rhizosphere microbe containing sulfate-dissimilated reduction genes. **a** Rhizosphere microbiome metabolic pathways associated with the S-cycling in wild and cultivated rice. Red arrows represent the dominant pathways in wild rice rhizosphere, while blue arrows in cultivated. Bold genes represent significant difference, but not in reverse. **b** Differences in metabolic pathways between dissimilatory and assimilatory sulfate reduction pathways. Red arrows represent the dissimilatory sulfate reduction which is the dominant pathway in wild rice rhizosphere, and blue arrows represent the dominant pathway in cultivated. In the histogram, the red columns represent the rhizosphere gene abundance in wild rice, while the blue columns represent the cultivated. **c** Families containing dissimilatory sulfate reduction genes (*dsrAB*, *aprAB*). **d** Colony morphology of isolated microbe. **e** PCR amplification of dissimilatory sulfate reduction genes (*dsrA*, *aprA*)

Differences of metabolic pathways in rhizosphere between wild and cultivated rice were sorted out against the KEGG database (Fig. 3a). The wild rice rhizosphere had higher gene abundance during the processes of dissimilatory sulfate reduction (*aprAB*, *dsrAB*), tetrathionate reduction (*ttrABC*), elemental sulfur disproportionation (*sor*) and reduction (*sreAB*). The rhizosphere of cultivated rice were involved in assimilatory sulfate reduction (*cysDHIJK*, *cysNC*, *SIR*, *metBZ*), sulfate and thiosulfate transport (*cysAPWU*), thiosulfate decomposition (*soxABCXYZ*, *glpE*, *phsABC*), and sulfide dehydrogenation reaction (*fccAB*). Based on the comparison of functional gene abundance in S-cycling, it was found that sulfate reduction exhibited different metabolic trends in wild and cultivated rice, which utilized the same substrate (Fig. 3b). The wild rice rhizosphere microbe was focused on dissimilatory sulfate reduction which produced hydrogen sulfide discharged into the soil, and the microbiome in cultivated rice rhizosphere tended to assimilatory sulfate reduction which formed sulfur-containing amino acids for their life activities. Therefore, dissimilatory sulfate reduction which consists of two main steps (*aprAB, dsrAB*) was selected as the process for our study.

### Selection, Isolation and Verification of Functional Microbe

Given the feasibility of synthetic microbiome rebinding, isolated microbe in wild rice rhizosphere should dominate in abundance and correlation. To reproduce the characteristics of wild rice in cultivated rice rhizosphere, *Comamonadaceae* and *Rhodospirillaceae*, two families containing *aprA* and *dsrA* genes respectively, were selected to construct the synthetic microbiome for subsequent verification (Fig. 3c). Four *Comamonadaceae* strains, including *Ideonella sp.*, *Acidovorax sp.*, *Acidovorax wautersii*, *Paracidovorax avenaep*, and two *Rhodospirillaceae* strains, including *Azospira sp.*, *Azospirillum sp.*, were obtained from the rhizosphere soil of wild rice by high-throughput isolation, culture and identification (Zhang et al. 2021) (Fig. 3d). PCR amplification was used to confirm the isolated strains contained *aprA* and *dsrA*, respectively (Fig. 3e).

### Construction and Effect Verification of the Synthetic Microbiome

**Fig. 4:**
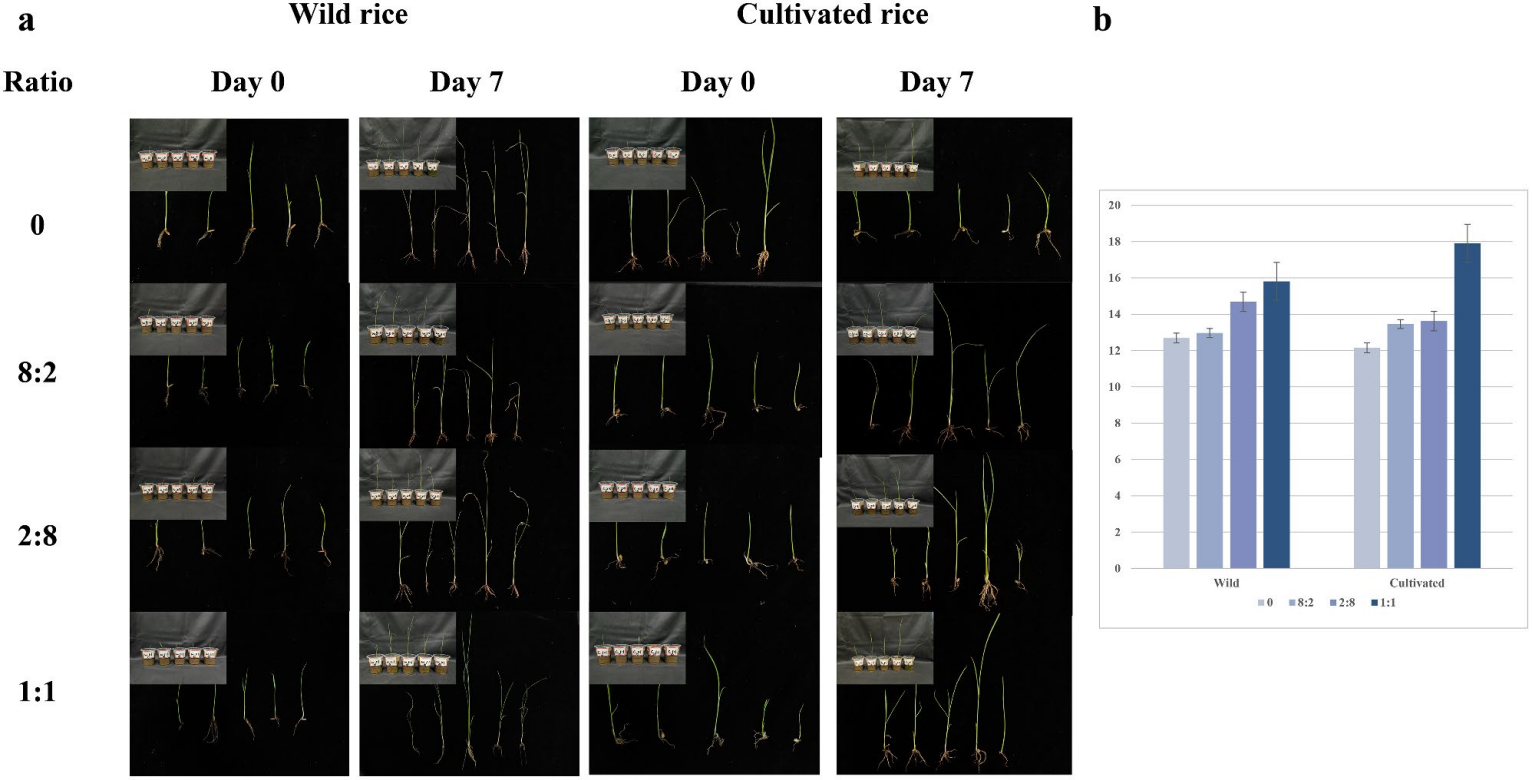
Response of rice roots to synthetic microbiome. **a** Root and pot figures of wild and cultivated rice at day 0 and day 7 under different synthetic microbiomes inoculation. There are 5 pots as biological replicates for each treatment. **b** Difference of root elongation (/mm) (One-way ANOVA, *p* < 0.01)

To access the dissimilatory sulfate-reducing ability of the isolated strains containing two key step genes (*dsrA*, *aprA*), respectively, synthetic microbiomes were constructed with two families in three ratios (8:2, 1:1, 2:8). The function genes *aprA* and *dsrA* are involved in dissimilatory sulfate reduction to produce hydrogen sulfide, which stimulates root growth at low concentrations. Therefore, comparing the effect of three kinds of synthetic microbiomes with the blank control group, root elongations were measured on day 0 and day 7 (Fig. 4a).

In order to confirm the characteristics of the constructed synthetic microbiome in the rhizosphere of wild rice, three types of synthetic microbiomes were added into the gamma-ray sterilized soil, and wild rice was transplanted at seedling stage for 7 days.

The results of one-way ANOVA (Fig. 4b) showed that the root elongation of wild rice in 1:1 and 2:8 synthetic microbiome treatment groups were significantly higher than that in blank control group, while the effect of 1:1 was better than that of 2:8. It was concluded that the constructed synthetic microbiome can promote rice root elongation, which may be due to the production of hydrogen sulfide during microbial activity.

To evaluate the effect of the synthetic microbiome on cultivated rice under natural conditions, three types of synthetic microbiomes were added into the natural soil, and cultivated rice was transplanted at seedling stage for 7 days.

According to the results of one-way ANOVA (Fig. 4b), the root elongation of the three synthetic microbiome groups were significantly higher than that of the blank control group, and the effect sequence was 1:1, 2:8, 8:2. Due to the promoting effect of hydrogen sulfide produced in the process of dissimilatory sulfate reduction on the elongation of rice root, it might suggest that the inoculation of synthetic microbiome could enhance the dissimilatory sulfate reduction in rice rhizosphere. It was concluded that the constructed synthetic microbiome might reproduce the S-cycling characteristics of wild rice in the cultivated rice rhizosphere.

## Discussion

Rice (*Oryza sativa L*.) serves as a staple food source for nearly half of the global population (Zeng et al. 2017). Enhancing rice production is a crucial strategy for promoting worldwide food security. Sulfur (S), as one of the main components of protein, plays a crucial role in determining rice yield and grain quality (Qiao et al. 2023). Consequently, we hope to alter rhizosphere sulfur cycling by artificially regulating rhizosphere microbiome and then influencing sulfur utilization by plants.

In present study, culture-independent technique-shotgun metagenomics sequencing was applied to assess the response of S-cycling function and communities to rice domestication, particularly focusing on pathways of sulfate reduction. Our study supported the differences in microbial community composition and function between wild and cultivated rice rhizosphere. According to the PCA plot and the alpha diversity chart (Fig. 1), it can be initially inferred that there are significant differences in the composition of the microbial community in the rhizosphere between wild rice and cultivated rice. From the relative abundance (Fig. 2a), it can be found that the microbial groups with high abundance are similar, which may be caused by environmental factors such as soil conditions and climate. More important are those that the microbial communities lost in domestication may not be present in large numbers but still play a significant role (Chang et al. 2021). In the correlation network (Fig. 2b–i), wild rice contains more nodes that are not involved in the S-cycling. Since it is based on the correlation analysis initiated by the microbial groups involved in the sulfur cycle, the more green nodes means that the microbial communities in the wild rice rhizosphere are more closely related to each other and are not even limited by functional divisions. In addition, the sudden decrease in the correlation network index formed from the aggregated data of wild rice may indicate that there are significant differences in the key functional microbial groups of the S-cycling recruited from the rhizosphere of different wild rice species. Different species of wild rice may utilize different microbiomes to achieve a similar goal.

Based on the S-cycling metabolic pathways in the rhizosphere of wild and cultivated rice (Fig. 3a), it can be confirmed there are significant differences in the tendency of S-cycling function. We found some interesting trends in the process of sorting out metabolic pathways. Firstly, take sulfate reduction as an example, wild rice rhizosphere is more focused on dissimilatory sulfate reduction to produce H_2_S which mediates multiple biological processes in plants, particularly in abiotic stress response (Arif et al. 2021). But the cultivated focus much more on the assimilatory sulfate reduction to retain sulfur in themselves. Secondly, thiosulfate, has a considerable role in plant resistance to pests, also can be directly absorbed by plants (Nakajima et al. 2019). Compared with cultivated rice, more thiosulfate was formed in wild rice rhizosphere, but less was decomposed. We suggest that wild rice rhizosphere microbe may emit a certain amount of thiosulfate into plant rhizosphere due to the difference between composition and decomposition. Thirdly, the cultivated rice rhizosphere has stronger function on transport of sulfate and thiosulfate, which are directly absorbed and utilized by plants (Kopriva et al. 2019). It means that rhizosphere microbiome in cultivated rice shows stronger competition with plants for nutrients. Finally, regarding elemental sulfur, wild rice rhizosphere showed a stronger ability to utilize it, while cultivated rice rhizosphere microorganisms produced more sulfur that could not be utilized by plants, even be harmful (Fuentes-Lara et al. 2019). Based on the above results, it is concluded that microbiome in the wild rice rhizosphere are more prone to interact with plants. In other words, domestication reduces beneficial interactions between plants and rhizosphere microbes, consistent with the idea of Raaijmakers and Kiers (2022).

In inoculation test, the synthetic microbiome had a significant impact on rice root elongation, which might be due to the change in rice sulfur utilization (Fig. 4b). However, future studies need to determine the H_2_S content to confirm it.

To determine whether the function of the rhizosphere microbiome can be manipulated by regulating its composition, we arranged inoculation validation of the synthetic microbiome. In our research, members of the synthetic microbiome each adopted a portion of the destination pathway, making the target function play accurately (Fig. 3b–c). In tests of mixed with different proportions, the function of equal proportion mixing plays a best effect (Fig. 4b). This may be due to the same proportion of constructed synthetic microbiome, which involves equal amounts of functional genes involved in the two stages of dissimilatory sulfate reduction. Compared to inoculated synthetic microbiome with proportion differences, there are less limitations in functional performance.

In our results, we had achieved changes in the propensity of S-cycling metabolic pathways in rice rhizosphere. Therefore, we believe that step-by-step constructing synthetic microbiome based on metabolic pathway function genes may be an effective scheme to effectively promote the implementation of synthetic microbiome. Conversely, we can also manipulate the structure of microbial community in rhizosphere by inoculating the synthetic microbiome, thereby inhibiting the progress of certain pathways. For example, when we increase the functional microbe in one stage of a specific pathway, the microbe could potentially compete with functional microbes involved in other stages, thereby reducing their efficiency. Consequently, this competition could limit the overall operation of the entire pathway.

In this research, we confirmed the feasibility of the approach to improve sulfur utilization in cultivated rice by inoculating microbiomes obtained from wild rice rhizosphere into cultivated rice rhizosphere. It has a good potential to use the mixed cultivated rice and wild rice to improve the final microbial function of cultivated rice, which needs more information such as the number of each kind of plants as well as the different growing stages accordingly. It will need more detection to provide the final conclusion as the next step for the current research.

## Conclusion

In summary, during domestication, the composition of S-cycling microbial community in rice rhizosphere tended to be consistent and the diversity decreased, and the mutually-beneficial relationship between S-cycling microbial community and host was weakened. The inoculation of the synthetic microbiome had a significant impact on root elongation of rice, which might be due to the change in rice sulfur utilization. This study highlights the response of S-cycling microbiome in rice rhizosphere to domestication, and based on this, proposes a synthetic microbiome construction scheme to improve rice sulfur utilization. The step-by-step construction of synthetic microbiome based on functional genes may represent a promising target for promoting synthetic microbiome to achieve the development of sustainable agriculture.

### Electronic Supplementary Material

Below is the link to the electronic supplementary material.

Supplementary Material 1

## Data Availability

The raw and processed metagenomic data are available on NCBI and affiliated with bioproject number PRJNA632564.
