# Supplementary material for: A Synthetic Microbiome Based on Dominant Microbes in Wild Rice Rhizosphere to Promote Sulfur Utilization
Source: Rice (N Y). 2024 Mar 1;17:18. doi: 10.1186/s12284-024-00695-y (PMC10907558; doi:10.1186/s12284-024-00695-y)
Supplement: Supplementary file 1 — Supplementary Material 1 [file 12284_2024_695_MOESM1_ESM.docx]

Supplementary table 1 Compositions of postgate medium (liquid)

|  | g/L |
| --- | --- |
| KH₂PO₄ | 0.5 |
| NH_4_Cl | 1.0 |
| Na_2_SO_4_ | 1.0 |
| CaCl_2_ | 0.05 |
| MgCl₂·6H₂O | 2.0 |
| Yeast Extract | 1.0 |
| Ascorbic Acid | 0.1 |
| Sodium Thioglycolate (C_2_H_3_NaO_2_S) | 0.1 |
| FeSO₄·7H₂O | 0.5 |
| Sodium D-lactate (C_3_H_5_NaO_3_) | 1.1 |

Tips: Postgate medium (Solid) - add 20g/L AGAR in postgate medium (liquid)
